# Fast optoelectric printing of plasmonic nanoparticles into tailored circuits

**DOI:** 10.1038/srep46506

**Published:** 2017-04-13

**Authors:** José A. Rodrigo

**Affiliations:** 1 Universidad Complutense de Madrid, Facultad de Ciencias Físicas, Ciudad Universitaria s/n, Madrid 28040, Spain

## Abstract

Plasmonic nanoparticles are able to control light at nanometre-scale by coupling electromagnetic fields to the oscillations of free electrons in metals. Deposition of such nanoparticles onto substrates with tailored patterns is essential, for example, in fabricating plasmonic structures for enhanced sensing. This work presents an innovative micro-patterning technique, based on optoelectic printing, for fast and straightforward fabrication of curve-shaped circuits of plasmonic nanoparticles deposited onto a transparent electrode often used in optoelectronics, liquid crystal displays, touch screens, etc. We experimentally demonstrate that this kind of plasmonic structure, printed by using silver nanoparticles of 40 nm, works as a plasmonic enhanced optical device allowing for polarized-color-tunable light scattering in the visible. These findings have potential applications in biosensing and fabrication of future optoelectronic devices combining the benefits of plasmonic sensing and the functionality of transparent electrodes.

Due to their peculiar photothermal and optical properties, plasmonic nanoparticles (NPs) have been actively exploited in a large variety of applications in science and technology^1^,^2^,^3^. Specifically, such NPs (e.g.: colloidal silver and gold particles) strongly scatter and absorb light near to their localized surface plasmon resonance (LSPR), and therefore, can be applied as subwavelenght light emitters or heat nanosources for lithography^4^,^5^ and photothermal therapy^6^,^7^, etc. An important fact is the dependence of the LSPR intensity and wavelength on the kind of metal, NP size and shape, as well as the dielectric constant of the surrounding medium. This capability to tune the plasmon resonance is crucial, for example, in the development of plasmonic detection of biomolecules (biosensing), particle-based therapies, nanoantennas, enhanced Raman spectroscopy, etc. Further development of plasmonic applications relies on the emergence of new fabrication methods of plasmonic devices, which are often fabricated by using expensive e-beam lithography methods.

In the last years, optical tweezers (point-like laser traps) have opened the door to a cost-effective fabrication method based on deposition of metal NPs onto substrates with a positional precision of tens of nanometers^8^,^9^,^10^,^11^. In this case the optical trapping forces are applied to immobilize NPs (captured from the colloidal solution) one by one on specific locations of the substrate. This is often known as *optical or laser printing* because the laser trap brings the particle close enough to the substrate (glass coverslip) so that the attractive force between them dominates resulting into a fixed NP. Specifically, the laser helps the particles overcome the electrostatic repulsion and then be attached to the substrate via van der Waals attraction^8^,^9^,^10^,^11^. The chemical and electrostatic properties of the substrate play a crucial role in the particle attachment. In practice, the glass coverslip has to be chemically treated to tune its surface charge to both obtain particle printing and avoid spontaneous deposition^10^,^11^.

On the other hand, transparent conductive indium tin oxide (ITO) substrates are currently the premier choice to fabricate transparent electrodes in a large variety of optoelectronic devices including liquid crystal displays, touch screens, and organic light-emitting devices. Thus, deposition of metal NPs onto ITO substrates^12^,^13^ could play an important role in creating future plasmonic and optoelectronic devices.

This work presents an alternative strategy to achieve fast selective deposition of plasmonic NPs onto ITO substrates along tailored circuit shapes. It is based on an innovative optoelectric patterning technique that allows for massive light-induced electrophoretical deposition of metal NPs along arbitrary two-dimensional (2D) curves. Specifically, a polymorphic laser beam that can be shaped in the form of arbitrary curve^14^,^15^ is strongly focused onto the ITO substrate in presence of an uniform electric field to achieve optoelectric printing of the metal NPs. The laser curve determines the circuit’s shape while the printed NPs form subwavelength assemblies working as light scatters/emitters or absorbers for resonant wavelengths. We experimentally demonstrate that printed circuits of silver NPs (spheres of 40 nm with LSPR at 415 nm, Sigma Aldrich 730807) exhibit a rich spectral response in the visible range that can be tuned as a function of the polarization of the incoming light.

Interestingly, under white light illumination, the printed circuit works as a polarized-color-tunable plasmonic light scatter. This is caused by multiple subwavelength assemblies of silver NPs (ranging from simple dimers to chains and clusters of several NPs) that strongly scatter light according to polarization dependent plasmon resonance modes. Here, we have considered silver NPs because they exhibit efficient LSPR being widely used in the fabrication of plasmonic devices. For example, it has been found that gold nanorods are recommended for optical plasmon imaging while silver nanorods are more efficient in plasmon sensing^16^. Nevertheless, the developed optoelectric technique can be applied for any type of plasmonic NPs.

## Results

### Principle of the technique

**Figure 1:**
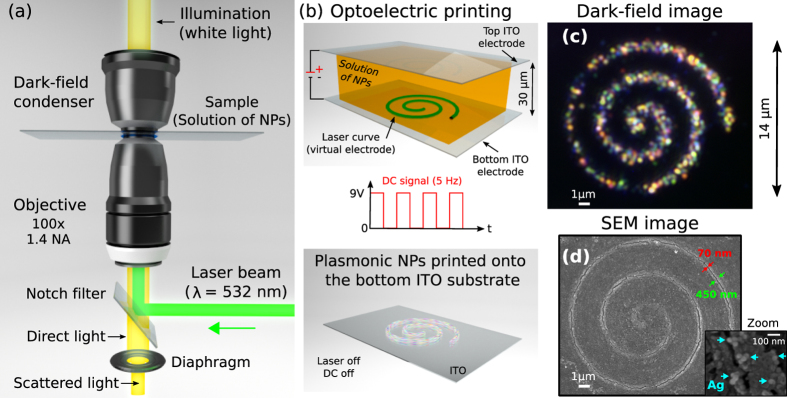
(**a**) Sketch of the experimental setup (inverted dark-field microscope). (**b**) The solution of silver NPs was enclosed between two ITO coverslips electrodes required for optoelectric printing. (**c**) Color dark-filed image that shows the light emitted by the silver NP assemblies created along an Archimedean spiral circuit, whose SEM image is displayed in (**d)**. Zoom inset in (**d)** shows several subwavelength NP assemblies (≤150 nm). The width of laser-heated region (~450 nm) is similar to the laser curve’s one.

The proposed optoelectric printing technique exploits a laser beam strongly focused in the form of diffraction-limited curve^14^,^15^ to create a laser-heated printing region onto the targeted ITO substrate in presence of an uniform electric field, which is created by applying a direct current (DC). Note that the same microscope objective lens focusing the laser beam onto the ITO substrate is used to image the NPs under dark-field illumination, see the inverted dark-field microscope configuration sketched in Fig. 1(a). When the DC electric field is activated the laser-heated region works as a *virtual light*-*activated electrode* able to massively capture and attach the NPs exclusively onto the ITO substrate illuminated by the laser curve, under specific conditions of the applied DC electric field and optical power. Indeed, the polarity of the DC has to be applied taking into account the charge of the NPs in order to prevent undesired spontaneous deposition of the NPs onto the targeted ITO substrate. In our case, we have considered silver NPs (in colloidal dispersion, aqueous buffer containing sodium citrate as stabilizer, Sigma Aldrich 730807) that are negatively charged due to the capping citrate agent used to prevent them from aggregating. Thus, in our case, the DC polarity is such that the targeted bottom ITO-coated glass coverslip is negatively charged while the top one enclosing the solution of NPs results positively charged, see Fig. 1(b).

In these conditions, selective optoelectric printing of NPs is initiated when the light curve is projected on the bottom ITO electrode and simultaneously applying a pulsed DC electric field (e.g., low frequency square wave of 5 Hz) by using a simple 9 V battery as power source. The laser-heated region warms the liquid, resulting in a temperature gradient, that in turn induces fluid convection as well as gradients of its electrical permittivity and conductivity. This, in presence of the applied electric field, strongly attracts the NPs to the illuminated region (*virtual* electrode) where they result permanently attached. For example, Fig. 1(c) shows an Archimedean spiral circuit of silver NPs printed in a time of about 2 s. Let us underline that the deposition of NPs along the illuminated region (curved circuit) stops in the absence of the DC electric field. Thus, a pulsed DC signal results useful for the control of the deposition process needed to achieve uniform printing of the NPs along the curve and avoid possible overheating effects in the illuminated region. Note that in the absence of the DC electric field, selective deposition of NPs along the curve could be achieved by exploiting the conventional optical printing mechanism^8^,^9^,^10^,^11^. In such a case, it consists of using the light radiation pressure force^8^,^9^,^10^,^11^ to push the NPs against the top ITO substrate when the curved-laser beam is focused on it instead of the bottom one. Finally, when the applied DC electric field is constant (not pulsed) and its polarity is inverted, thus the targeted bottom ITO electrode is positively charged, the silver NPs resulted permanently attached to the whole bottom ITO surface, see Supplementary Information (Fig. S1).

Note that the proposed technique is completely different from the optolectronic tweezers based on optically induced dielectrophoretic (DEP) forces that require a dielectric photoconductive substrate fixed to the ITO electrode (limiting its implementation in device fabrication) as well as applying high alternate current (AC) electric field of about 100 kV/m with frequencies above 100 kHz, see for example^17^,^18^,^19^. Optolectronic tweezers were conceived as an alternative to the well-known laser tweezers for manipulation/sorting of numerous dielectric particles and have been recently applied for assembling ions and nanomaterials onto the photoconductive substrate^20^. The use of bare ITO electrodes (without photoconductive substrate) for optolectronic tweezers has been recently demonstrated for manipulation and deposition of carbon nanotubes^21^, but it also requires high AC electric field (100–140 kV/m) with frequency 10–100 kHz. In contrast, the proposed technique permits selective light-driven deposition of metal NPs onto a bare ITO electrode by using a low DC electric field that can be applied by using a simple 9 V battery. This is possible because the considered diffraction-limited laser curve exhibits high intensity gradients that allow for fast and selective heating of the illuminated ITO substrate when the DC electric field is activated, as demonstrated in the next Section.

Let us underline that this kind of diffraction-limited laser curve has been applied as an all-optical 3D manipulation tool for confinement and transport of dielectric micro-particles^14^,^15^ as well as metal NPs^22^ along arbitrary trajectories in a programmable way. These capabilities provide relevant advantages and practical versatility^22^, however, here the laser curve has been only applied for selective heating of the ITO substrate in presence of the DC electric field in order to achieve fast and permanent deposition of the NPs along the curve.

### Experimental results

Here, we have considered a laser beam with an optical power of ~40 mW (measured at the back aperture of the objective) and wavelength of 532 nm for which the ITO-coated coverslip (~12 Ohms/Sq) is mostly transparent (~80%), according to the manufacturer data (Diamond Coatings). The deposition of silver NPs is controlled by the *printing* time in which the DC electric field and laser curve are simultaneously activated. Specifically, we have applied a direct current of 590 mA (voltage of 9 V) given in pulses of 200 ms of duration (5 Hz square wave DC signal, 50% duty cycle). As it has been previously mentioned, Fig. 1(c) shows an Archimedean spiral circuit of silver NPs printed in a time of about 2 s when the DC electric field polarity is such that the bottom and top ITO electrodes resulted negatively and positively charged, respectively. This dark-field image demonstrates well-defined optoelectric printing of the NPs along the curve (onto the targeted bottom ITO electrode) and it has been obtained with white-light illumination when both the laser curve and DC electric field are deactivated, see Methods.

The scanning electron microscope (SEM) image displayed in Fig. 1(d) shows the printed spiral circuit onto the ITO substrate. This presents subwavelength cracking (width of 50–150 nm) along the spiral that has been created by mechanical stress due to the temperature gradients in the laser-heated region when the DC electric field was applied. The generated assemblies of silver NPs have a size of ~150 nm as the ones displayed in the zoom inset of Fig. 1(d). These subwavelength assemblies but also dimers and single silver NPs have been deposited in a laser-heated region of width ~450 nm, see Fig. 1(d), which is similar to the width of the laser curve. Similar results have been also obtained by applying a pulsed (5 Hz) DC signal of 4.5 V (490 mA) but it required about ×2 of optical power. These facts confirm the described light-induced electrophoretical deposition and assembling of silver NPs along the curve. Nevertheless, further research is required to completely determine the required threshold of the DC electric field given as a function of the optical power.

**Figure 2:**
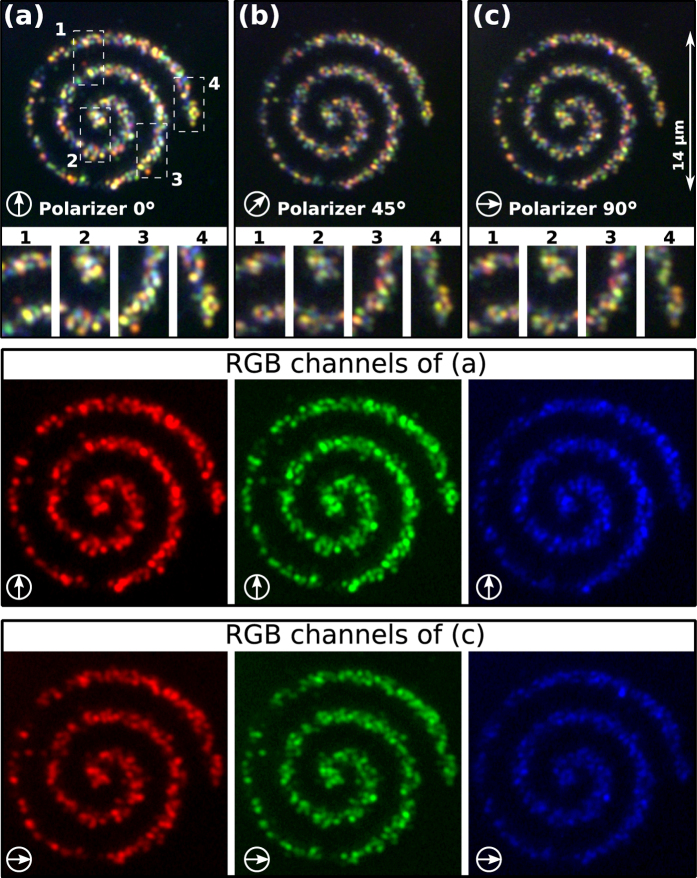
(**a–c**) Polarized-color-tunable behavior of the light scattered by the silver NP assemblies, observed when rotating a linear polarizer, see also Supplementary video 1. The RGB color channels for vertical and horizontal polarization are also displayed.

A single silver NP of 40 nm (sphere) illuminated with white light appears as a bright blue point source scatterer due to its LSPR centered at 415 nm. While, the size and geometry of silver NP assemblies determine their polarization-dependent spectral response. The rich spectral response (visible range) observed in Fig. 1(c) indicates the large variety of subwavelength silver NP assemblies printed along the circuit, as expected. Specifically, the printed circuit behaves as a polarized-color-tunable plasmonic light scatter (*emitter*). This can be easily demonstrated by rotating a linear polarizer (Thorlabs, LPVISB100-MP) placed in front of the color camera (Thorlabs, DCC1240C). Indeed, the dark-field images displayed in Fig. 2 demonstrate the polarized-color-tunable behavior of the silver NP assemblies as a function of the rotation angle of the polarizer, see also Supplementary video 1. As an example, the zoom insets of Fig. 2 show four parts of the spiral circuit where the color and shape of the scattered light vary when the polarizer rotates: Vertical linear polarization is transmitted in the case of Fig. 2(a), while in Fig. 2(b) and (c) the polarizer was rotated at 45 and 90 degrees, respectively. The observed change in the shape and color of the scattered light is explained by the polarization dependent surface plasmon resonance modes of the generated NP assemblies. Indeed, the relative peak intensities of such resonance modes can be successfully tuned by polarized excitation. This anisotropic response has been also observed in the case of a single gold nanorod^23^,^24^,^25^ and metal nanoprisms^26^. A complete characterization of the polarization and spectral properties of the scattered light from silver NP assemblies can be performed by using specialized dark-field spectroscopy/polarimetry techniques^25^,^26^, which is out of the scope of this work.

The latter results confirm that the generated assemblies of silver NPs along the circuit behave as polarization-controlled colorimetric nanostructures. This fact together with the LSPR peak shift as a function of the surrounding medium make attractive such *rainbow* NP assemblies for biosensing applications or tunable light scatters (emitters) at the micro/nano-scale. This kind of nanostructure is an alternative to complex-shaped NP such as nanorods, nanoprisms and nanostars that have found increased interest for applications such as optoelectronic devices and sensors due to their anisotropic responses^23^,^24^,^25^,^26^,^27^,^28^. Many of these applications require tailored deposition of such NPs onto the substrate in order to make full use of these anisotropies and rich spectral responses.

**Figure 3:**
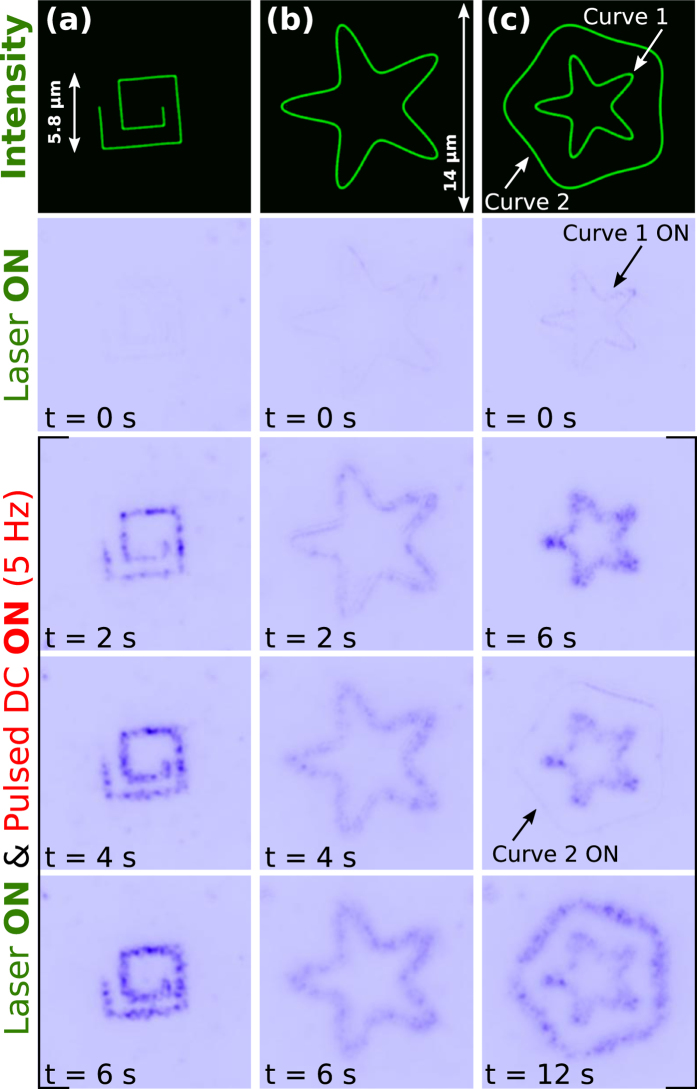
Progressive optoelectric printing of different circuits: (**a)** squared spiral, (**b)** starfish and (**c)** pentagonal circuit encircling a small starfish one. The corresponding laser curves are displayed in the top row, while the bottom rows (monochrome dark-field images) show the progressive deposition of silver NPs along the circuits when both the laser curve and pulsed DC signal are activated, see Supplementary video 2, 3 and 4.

In our case, tailored deposition of silver NPs can be achieved by exploiting the capability of reconfiguring the shape of the laser curve and the printing time. For example, Fig. 3 shows progressive optical printing for three circuits of different complexity that exhibit multiple corners and change of direction. As in the case of the Archimedean spiral circuit, the squared-spiral circuit of Fig. 3(a) presents well-defined deposition of silver NPs along the curve for a printing time of *t* = 2 s, as observed in the monochrome dark-field image (acquired by a high speed sCMOS camera: Hamamatsu, Orca Flash 4.0). The number of NP assemblies can be progressively increased as observed in Fig. 3(a) for *t* = 4 s and *t* = 6 s, see also Supplementary video 2. This optoelectric printing process occurs even in the corners of the curve of the squared-spiral circuit as well as in the starfish-shaped and polygonal circuits displayed in Fig. 3(b) (Supplementary video 3) and Fig. 3(c) (Supplementary video 4), respectively. To illustrate the versatility of the proposed technique for printing complex structures, e.g. by using two (or more) laser curves, the pentagonal circuit was created encircling a starfish one, see Fig. 3(c) and Supplementary video 4. Note that the described printing process is achieved when both the DC electric field and laser curve are simultaneously activated as demonstrated in Fig. 3 and Supplementary video 2,3,4.

**Figure 4:**
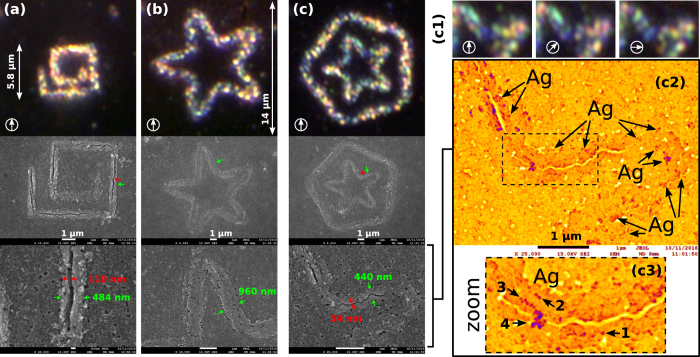
Dark-field and SEM images of the printed plasmonic circuits: (**a)** squared spiral, (**b)** starfish and (**c**) pentagonal circuit encircling a small starfish one. Dark-field images in c1 show the polarized-color tunable response of the subwavelength NP assemblies observed in the SEM image c2. The arrows in zoom inset c3 indicate silver NPs deposited in the form of monomer (1), dimer (2), chain of 6 NPs (3), and a cluster of several NPs (4).

The total printing time in the previous examples was 6 seconds for each curve. Figure 4 displays the corresponding color dark-field images (top row) and SEM images (bottom row) of the printed circuits. In the case of the squared-spiral circuit the laser-heated region has a width of ~484 nm similar to laser curve width. The observed crack along such a spiral has a width of ~110 nm indicating the mechanical stress effects caused by the temperature gradients during the optoelectric printing process, as it was previously discussed. Interestingly, the width of the laser-heated region can be easily increased, if needed, just by slightly defocussing the light curve onto the substrate. This is demonstrated in the case of the starfish circuit displayed in Fig. 4(b), where the width of the laser-heated region is about 960 nm while preserving well-defined printing of NPs and avoiding cracking. Such a laser-heated region is about ×2 bigger than the one (of ~440 nm) created when using a well focused star-shaped laser curve as shown in Fig. 4(c). The SEM image displayed in the bottom row of Fig. 4(c) shows subwavelength cracks of 50–100 nm, as in the case of the spiral circuits. Nevertheless, such subwavelength cracks in the ITO surface do not significantly affect to the printed circuit of silver NPs.

The dark-field images displayed in the zoom insets of Fig. 4(c1) show the polarization-controlled colorimetric response of the silver NP assemblies observed in the SEM image displayed in Fig. 4(c2) and zoom inset Fig. 4(c3). These results confirm the deposition, in the laser-heated region (width of ~400 nm), of single silver NPs as well as assemblies ranging from simple dimers/chains to subwavelength clusters of several NPs, as the ones indicated by the arrows in Fig. 4(c3). The geometry of such assemblies is responsible of the anisotropic response observed in Fig. 4(c1), as it has been previously discussed.

## Discussion

The developed optoelectric printing technique allows for fast (~2 s) and progammable deposition of numerous colloidal NPs onto a transparent electrode, along arbitrary 2D circuits that can be easily designed according to the considered application. This could be promising for printing plasmonic structures based on assembling of colloidal metal NPs such as silver and gold. The experimental results confirm that arbitrary curve-shaped circuits, printed by using silver NPs (spheres of 40 nm), can work as polarized-color-tunable light scatters in the visible due to multiple subwavelength assemblies of NPs created during the optoelectric deposition process. The color and polarization of the scattered light can be tuned by resonant polarized excitation of the silver NP assemblies under white light illumination. This fact together with the dependence of the LSPR intensity and wavelength on the dielectric constant of the surrounding medium (e.g.: biological fluid) have potential applications in biosensing but also in the fabrication of tunable light scatters (*emitters*) at micro/nano-scale. A polarized-tunable behaviour in the near-infrared range could be achieved by using larger assemblies of NPs or other types of particles such as rod and star NPs often used in plasmonic biosensing^23^,^24^,^25^,^26^. Note that, if needed, the Ag NPs could be coated in order to prevent oxidation or to improve the surface-plasmon-to-hot-electron conversion efficiency^30^.

Apart from such polarized-tunable light scatters, the printed circuits could be also applied as structurally tailored heat sources for plasmonic enhanced photo-thermal applications. Another advantage is that the NPs are printed onto an ITO substrate providing inherent versatility and electrical signaling capabilities. Other types of transparent electrodes can be also applied. The proposed technique is envisioned to assist the fabrication of optoelectronic devices combining the benefits of plasmonic structures and the functionality of transparent electrodes.

## Methods

### Experimental setup and sample preparation

A programmable SLM (Holoeye PLUTO, pixel size of 8 *μ*m) was used to shape in real time a collimated laser beam (Laser Quantum, Ventus, *λ* = 532 nm, linearly polarized) into the considered laser curves, as reported in ref. 15. While, the microscope objective lens (Olympus UPLSAPO, 1.4 NA, 100X, oil immersion) strongly focused the laser curve onto the ITO substrate. To mitigate spherical aberration arising form the glass-sample refractive index mismatch, an oil immersion with refractive index *n* = 1.56 (Cargille Labs Series A) has been used^14^,^15^. The printed circuits of plasmonic NPs were observed with white light illumination (high power LED, SugarCube Ultra) under a dark-field microscope (oil immersion condenser Nikon, 1.43 NA), as sketched in Fig. 1(a). To study the optoelectric printing process in real time, the monochrome dark-field images of Fig. 3 were recorded by using a high speed sCMOS camera (Hamamatsu, Orca Flash 4.0, 16-bit gray-level, pixel size of 6.5 *μ*m). Note that a Notch filter (Semrock, dichroic beamspliter for 532 nm) redirected the laser beam into the objective lens, that prevents saturating the camera by backscattered laser light. This filter has been removed from the setup when observing the color dark-field images. The colloidal solution of silver NPs were filled into the sample cell directly from the aqueous solution provided by the manufacturer (Sigma Aldrich 730807). Each ITO coverslip (thickness 0.17 mm) has been electrically contacted by using an aluminum conductive tape (thickness ~30 *μ*m), far enough from the printing region in order to obtain an uniform electric field. Finally, the optoelectric process has been performed by using a customized Labview program for controlling both the SLM and the applied DC signal supplied by a digital micro-controller (Arduino Mega).

### Beam shaping technique

The laser curve has been created by focusing the polymorphic beam^15^:


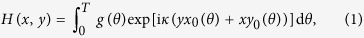


which was encoded as a hologram into the SLM. Specifically, *H*(*x, y*) can prescribe the intensity and phase of the laser beam along arbitrary curves, where:


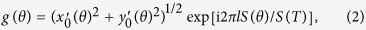


*κ* = 2*π*/*λ*f with f being the focal length of the focusing lens, and *T* = 2*π* for closed curves. The polar coordinates of the parametrized curve are *x*_0_(*θ*) = *R*(*θ*) cos *θ* and *y*_0_(*θ*) = *R*(*θ*) sin *θ*, with


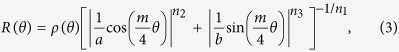


where the real numbers **q** = (*a, b, n*_1_, *n*_2_, *n*_3_, *m*) are the design parameters of the curve. In general, the function *ρ*(*θ*) is constant except, for example, in the case of spirals. The expression *R*(*θ*) is known as Superformula and it was found by J. Gielis^29^ in the study of biological and other natural forms: shapes of plants, micro-organisms (e.g.: diatoms), small animals (e.g.: starfish), crystals, etc. The use of the Superformula in *H*(*x, y*) provides a practical and versatile way to create a large variety of beam shapes^15^. In particular, the Archimedean and squared spirals correspond to *ρ*(*θ*) ∝ *θ* with **q** = (1, 1, 1, 1, 1, 0) and *ρ*(*θ*) ∝ *e*^0.2*θ*^ with **q** = (1, 1, 100, 100, 100, 4), respectively. While, the starfish and pentagonal (raspberry-like) curves in Fig. 3(b,c) correspond to **q** = (10, 10, 2, 7, 7, 5) and **q** = (1, 1, 4, 4, 4, 5), respectively. We have used 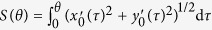 that sets a helical phase distribution making the laser curve a type of optical vortex of *topological charge l* (e.g.: *l* = 10 in the considered examples), see ref. 15 for further information. Finally, we underline that the integral *H*(*x, y*) has been numerically calculated (MATLAB) in about 10 seconds.

## Additional Information

**How to cite this article**: Rodrigo, J. A. Fast optoelectric printing of plasmonic nanoparticles into tailored circuits. *Sci. Rep.* **7**, 46506; doi: 10.1038/srep46506 (2017).

**Publisher's note:** Springer Nature remains neutral with regard to jurisdictional claims in published maps and institutional affiliations.

## Supplementary Material

Supplementary Video 1

Supplementary Video 2

Supplementary Video 3

Supplementary Video 4

Supplementary Information
