# Supplementary material for: Fast optoelectric printing of plasmonic nanoparticles into tailored circuits
Source: Sci Rep. 2017 Apr 13;7:46506. doi: 10.1038/srep46506 (PMC5390277; doi:10.1038/srep46506)
Supplement: Supplementary Information [file srep46506-s5.pdf]

Supplementary Information for:
"Fast optoelectric printing of plasmonic nanoparticles into tailored circuits"

José A. Rodrigo
Universidad Complutense de Madrid, Facultad de Ciencias Físicas, Ciudad Universitaria s/n, Madrid 28040, Spain

FURTHER INFORMATION ABOUT THE REPORTED OPTOELECTRIC DEPOSITION MECHANISM

The reported optoelectric patterning technique allows for straightforward deposition of plasmonic nanoparticles along curved-shaped circuits. In particular, we have considered Ag nanoparticles of 40 nm (in colloidal dispersion, aqueous buffer containing sodium citrate as stabilizer, Sigma Aldrich 730807) that have been deposited onto a transparent conductive indium tin oxide (ITO) substrate by using a strongly focused laser beam in form of diffraction-limited curve and by applying a low frequency pulsed DC electric field. Specifically, the Ag nanoparticles were deposited onto the bottom ITO-coated coverslip as indicated in Fig. 1(a)-(b) of the main text, by applying a DC of 590 mA (voltage of 9 V) given in pulses of 200 ms of duration (5 Hz square wave DC signal, 50% duty cycle). For example, Fig.1(c) shows an Archimedean spiral circuit of Ag nanoparticles printed in a time of about 2 s. To prevent undesired spontaneous deposition of the NPs onto the targeted ITO substrate, the polarity of the DC has to be applied taking into account the charge of the NPs. In our case, the Ag nanoparticles are negatively charged due to the capping citrate agent used to prevent them from aggregating. Thus, the applied DC polarity is such that the targeted bottom ITO-coated glass coverslip is negatively charged while the top one enclosing the solution of NPs results positively charged, see Fig. 1(b).

Here, we show that if the applied DC field is constant (not pulsed) and its polarity is inverted (thus the bottom ITO electrode results positively charged), then, the Ag nanoparticles result permanently attached to the whole bottom ITO substrate as observed in Fig.S1. This result demonstrates undesired massive non-selective deposition of nanoparticles illustrating the important role played by the DC polarity.

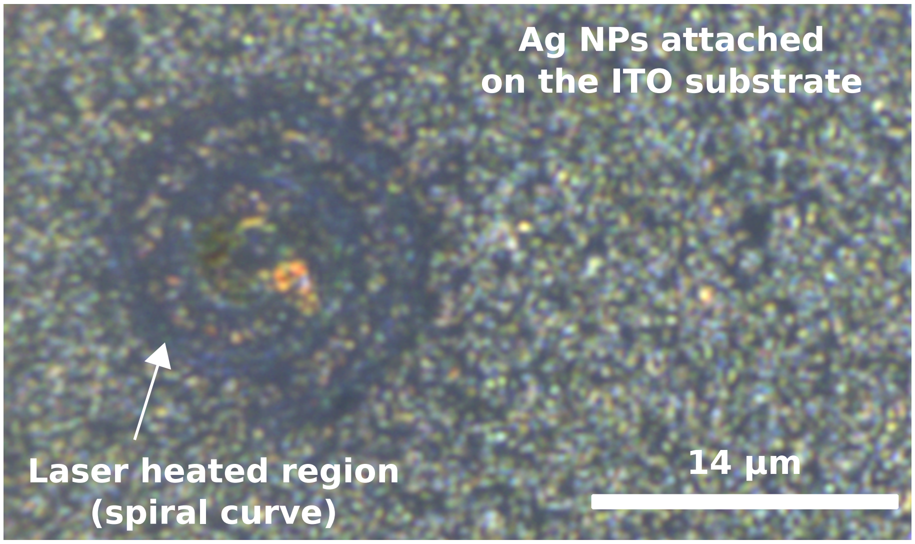

Figure S1. This dark-field image shows permanent massive deposition (non-selective) of Ag nanoparticles (spheres of 40 nm negatively charged due to capping citrate agent, Sigma Aldrich 730807) onto the ITO substrate, obtained when this electrode has been positively charged by applying a constant DC electric field (590 mA, voltage of 9 V) during ~2 s.

Video legends included in the supplementary information:

- Supplementary video 1: It shows the polarized-color-tunable response of the light scattered by silver NPs printed along an Archimedean spiral circuit displayed in Fig.1 and Fig.2.
- Supplementary video 2: Progressive optoelectric printing process of silver NPs into a squared-shaped spiral circuit (Fig.3a).
- Supplementary video 3: Progressive optoelectric printing process of silver NPs into a starfish-shaped circuit (Fig.3b).
- Supplementary video 4: Progressive optoelectric printing process of silver NPs into a complex circuit made by using two laser curves (Fig.3c).
